# Intraoperative antithrombotic drug removal during heart transplantation: A case series from the International Safe and Timely Antithrombotic Removal (STAR) registry^1^

**DOI:** 10.1016/j.jhlto.2025.100369

**Published:** 2025-08-20

**Authors:** Jan Schmitto, Filip De Somer, Matthias Thielmann, Nandor Marczin, Arjang Ruhparwar, Anna L. Meyer, Christian Hagl, Marijana Matejic-Spasic, Daniel Wendt, Weihong Fan, Efthymios N. Deliargyris, Robert F. Storey, Michael Schmoeckel

**Affiliations:** a Department of Cardiac, Thoracic, Transplantation, and Vascular Surgery, Hannover Medical School, Hannover, Germany; b Heart Centre, Ghent University Hospital, Ghent, Belgium; c Department of Thoracic, and Cardiovascular Surgery, West German Heart and Vascular Center, Essen, Germany; d Department of Anaesthesia and Critical Care, Harefield Hospital, London, United Kingdom; e Faculty of Medicine, Department of Surgery & Cancer, Imperial College, London, United Kingdom; f Department of Cardiac Surgery, University Hospital Heidelberg, Heidelberg, Germany; g Department of Cardiac Surgery, Klinikum Grosshadern, Ludwig-Maximilians-University, Munich, Germany; h German Centre for Cardiovascular Research (DZHK) Partner Site Munich, Munich, Germany; i CytoSorbents GmbH, Berlin, Germany; j Faculty of Medicine, University Duisburg-Essen, Essen, Germany; k CytoSorbents Inc., Princeton, New Jersey; l Division of Clinical Medicine, University of Sheffield, Sheffield, UK; m NIHR Sheffield Biomedical Research Centre, Sheffield Teaching Hospitals NHS Foundation Trust, Sheffield, UK

**Keywords:** orthotopic heart transplantation, direct-acting oral anticoagulants, hemoadsorption, bleeding, ticagrelor

## Abstract

**Background:**

Patients on heart transplant waiting lists are often on antithrombotic (AT) drugs. Emergency orthotopic heart transplantation (OHT) when performed on such patients without optimal washout periods increases the risk of severe perioperative bleeding. Intraoperative AT removal by hemoadsorption may mitigate excess bleeding risks.

**Methods:**

The international Safe and Timely Antithrombotic Removal (STAR) registry captures real-world outcomes (ClinicalTrials.gov# NCT05077124). Included patients were on ticagrelor or direct-acting oral anticoagulants (DOACs) undergoing emergent OHT. Hemoadsorption was integrated into the cardiopulmonary bypass (CPB) circuit. Bleeding was assessed with the universal definition of perioperative bleeding (UDPB) and volume of chest tube drainage (CTD).

**Results:**

Seven patients were included (3 ticagrelor, 2 apixaban, 2 dabigatran; mean age 39.1 ± 11.1 years; 4 females). Mean time from the last AT dose to surgery was 29.4 ± 13.4 hours. Mean CPB duration was 206.0 ± 56.9 minutes with a mean device flow of 340 ± 126 ml. There were no massive bleeding events (UDPB 4), surgical revisions to control bleeding, or deaths within 30 days. Severe bleeding (UDPB 3) occurred in 1/7 (14.3%). Mean 12-hour and 24-hour CTD were 385.7 ± 263.4 m and 586.1 ± 315.0 ml, respectively. No device-related adverse events were reported.

**Conclusions:**

This case series from the ongoing STAR registry shows that intraoperative AT removal is simple and potentially effective in minimizing serious perioperative bleeding in patients on ticagrelor or DOACs undergoing OHT. Prospective, controlled studies in larger cohorts are needed to validate these promising observations.

## Background

The timing of orthotopic heart transplantation (OHT) is unpredictable based on organ availability. Patients on the waiting list are often on antithrombotic drugs (AT) and when a heart becomes available, they proceed to emergent surgery without the option of washing out.^1^ Published reports have shown high rates of severe perioperative bleeding in patients on AT who undergo emergent cardiac surgery.^2^

A hemoadsorption device filled with biocompatible polymer beads has been shown to effectively remove some of the most prescribed AT drugs from blood and has received CE mark approval for intraoperative on cardiopulmonary bypass (CPB) use in patients on ticagrelor or rivaroxaban undergoing cardiac surgery with the goal of reducing perioperative bleeding complications.^3^, ^4^, ^5^

The international Safe and Timely Antithrombotic Removal (STAR) registry collects patient-level data on real-world use patterns and outcomes with the use of this hemoadsorption device for antithrombotic removal (ClinicalTrials.gov identifier: NCT05077124). Here, we report bleeding outcomes in a case series of patients on ticagrelor or direct-acting oral anticoagulants (DOACs) undergoing emergent OHT with intraoperative use of the device.

## Methods

### Registry design

STAR is an international registry collecting real-world data on patients treated with the hemoadsorption device for antithrombotic drug removal as part of their routine clinical care (only inclusion criterion). Exclusion criteria included baseline severe thrombocytopenia <50.000 µl, disseminated intravascular coagulation, heparin-induced thrombocytopenia, and device use for indications other than antithrombotic removal. Retrospective or prospective cases are eligible, the latter requiring informed consent. Clinical outcomes are collected for 30 days after the index procedure with a primary focus on perioperative bleeding. Safety is assessed by the collection of investigator-reported device-related adverse events.

### Device use

**Figure 1:**
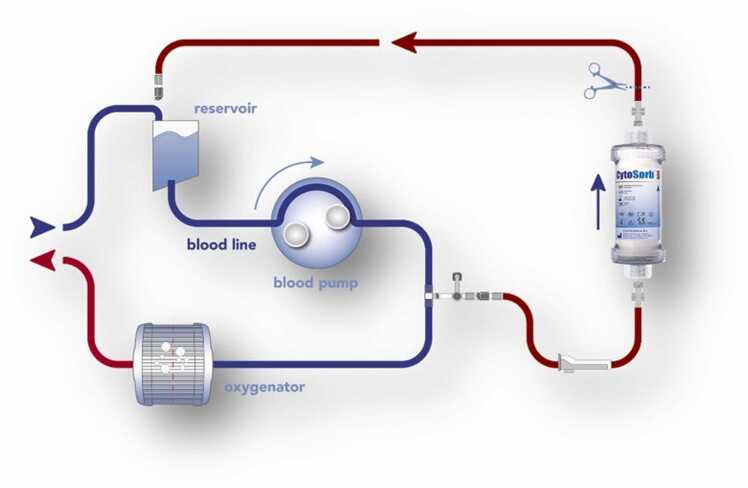
Integration of the adsorber into the heart-lung-machine.

Priming requires 2 liters of normal saline and can be completed in less than 5 minutes. The device can then be integrated as a shunt circuit in a standard CPB setup with a target flow rate of 100 to 700 ml/min (Figure 1).

### Outcome measures

Bleeding events were classified according to the universal definition of perioperative bleeding (UDPB) criteria. Additional outcomes included 24-hour chest tube drainage (CTD), blood product transfusions, reoperations for bleeding, and 30-day mortality. Serious bleeding complications were the primary focus of the analysis. The safety of the device was assessed by investigator-reported adverse events, including severity and related classifications.

### Statistics

Data were analyzed using SAS version 9.4 software (SAS Institute, Cary, NC). Continuous variables were expressed as mean with standard deviation (SD) or median with interquartile range, respectively. Categorical data were expressed as number of patients and frequencies.

### Ethical statement

This registry complies with the Declaration of Helsinki. National, central, or local approvals of respective ethics committees were granted for the STAR registry according to local regulations. Written consent was obtained from prospective patients and waived for retrospective patients.

## Results

### Baseline and operative characteristics

A total of 7 patients on ticagrelor or DOAC underwent emergent OHT with a mean time since last dose (TLD) of 29.4 ± 13.4 hours. Specifically, 3 patients were on ticagrelor with a mean TLD of 39.8 ± 9.5 hours (1 as monotherapy, 1 as DAPT with aspirin and 1 with warfarin), and 4 were on DOAC with a mean TLD of 21.6 ± 10.4 hours (2 on apixaban and 2 on dabigatran). Baseline characteristics are shown in Table 1. Mean age was 39.1 ± 11.1 years and 4 patients were female. Renal insufficiency was present in 4 patients, 2 of whom were on dialysis. Prior sternotomy had been performed in 4 patients (57.1%). The mean EuroSCORE-II was 7.6% ± 4.6%. All patients presented with poor or very poor left-ventricular ejection fraction. The preoperative coagulation and hematological profile is presented in Table 2.

**Table 1:** Demographics

Patient#	Age, years	Sex, female	BMI, kg/m^2^	ES-II, %	Previous cardiac surgery	NYHA class	Renal impairment, creatinine >2.2 mg/dl	Dialysis	IDDM	AT drug and half life	TLD (h)	ASA	Warfarin
1	35	1	25.4	6.5	1	III	0	0	1	15 mg dabigatran (12-17 h)	24.8	0	0
2	44	0	23.9	1.5	0	III	0	0	0	150 mg dabigatran (12-17 h)	14.1	0	0
3	51	1	30.6	3.8	0	III	1	0	0	5 mg apixaban (8-15 h)	12.6	0	0
4	41	1	35.7	13.8	1	IV	1	1	0	60 mg ticagrelor (8-12 h)	45.1	0	0
5	20	0	25.0	8.7	1	III	1	0	0	60 mg ticagrelor (8-12 h)	45.5	0	1
6	51	1	23.4	13.2	1	III	1	1	0	60 mg ticagrelor (8-12 h)	28.8	1	1
7	32	0	27.2	5.8	0	IV	0	0	0	5 mg apixaban (8-15 h)	35.9	0	0

**Table 2:** Preoperative Laboratory Measurements

Patient#	Platelet count (× 10^9^/liter)	Hemoglobin (g/dl)	APTT (sec)	INR	PTT (sec)	Fibrinogen (g/liter)
1	216	14.9	25.4	1.22	1	3.48
2	136	14.9	23.9	1.42	0	3.92
3	199	14.1	30.6	1.0	0	—
4	207	10.7	35.7	2.48	1	4.55
5	253	10.1	25.0	2.4	1	2.91
6	237	9.8	23.4	0.96	1	—
7	215	15.5	27.2	1.23	0	—

Abbreviations: APTT, activated partial thromboplastin time; INR, international normalized ratio; PTT, partial thromboplastin time.

The overall procedure time (skin-to-skin) was 6.9 ± 2.2 hours. The CPB and aortic cross clamping durations for the overall cohort were 206.0 ± 56.9 minutes and 100.1 ± 34.0 minutes, respectively. The mean hemoadsorption duration was 191.4 ± 52.3 minutes with a mean flow of 340 ± 126 ml through the hemoadsorption device.

### Clinical outcomes

No fatal or massive bleeding events (UDPB 4) occurred. One severe bleeding event occurred (UDPB 3) as a result of delayed sternal closure (which was not bleeding related). There were no reoperations required for bleeding control. Mean 12-hour and 24-hour CTD were 385.7 ± 263.4 ml and 586.1 ± 315.0 ml, respectively, with no patient experiencing >2,000 ml of CTD. None of the included patients received recombinant factor VIIa (rFVIIa, NovoSeven) and none of the patients with preoperative warfarin treatment received prothrombin complex concentrate. Perioperative transfusions are summarized in Table 3. Moreover, none of the patients received a reversal agent.

**Table 3:** Postoperative (Bleeding) Data

Postoperative data	N = 7
12-hour CTD, ml	385.7 ± 263.4
24-hour CTD, ml	586.1 ± 315.0
UDPB3	14.3% (1/7)
UDPB4	0% (0/7)
Revision for bleeding	0% (0/7)
Delayed sternal closure	14.3% (1/7)
Recombinant factor VII	0% (0/7)
Units of PRBC transfusion up to POD1	
5-10 units	14.3% (1/7)
2-4 units	42.9% (3/7)
1 unit	0% (0/7)
0 unit	42.9% (3/7)
Patients with PRBC transfusions up to POD1	57.1% (4/7)
Patients with PRBC transfusions anytime (until discharge) postoperatively	85.7% (6/7)
Units of PLT transfusion up to POD1	
5-10 units	0% (0/7)
2-4 units	14.3% (1/7)
1 unit	14.3% (1/7)
0 unit	71.4% (5/7)
Patients with PLT transfusions up to POD1	28.6% (2/7)
Patients with PLT transfusions anytime (until discharge) postoperatively	42.9% (3/7)
Units of FFP transfusion up to POD1	
5-10 units	14.3% (1/7)
2-4 units	42.9% (3/7)
1 unit	0% (0/7)
0 unit	42.9% (3/7)
Patients with FFP transfusions up to POD1	57.1% (4/7)
Patients with FFP transfusions anytime (until discharge) postoperatively	57.1% (4/7)

Abbreviations: CTD, chest tube drainage; FFP, fresh frozen plasma; PLT, platelets; POD, postoperative day; PRBC, packed red blood cells; UDPB, universal definition of perioperative bleeding.

Two patients with preoperative dialysis-dependent chronic renal failure underwent kidney transplantation the day after OHT. Survival at 30 days was 100%. No device-related adverse events were reported, and no primary graft dysfunction was observed.

## Discussion

Patients on ticagrelor or DOAC undergoing cardiac surgery before completing the recommended washout experience high rates of severe perioperative bleeding complications.^2^, ^6^ In the case of OHT, where surgery is universally emergent, washout of such drugs is not an option, and therefore a real unmet clinical need exists for strategies to reduce the high risk of severe perioperative bleeding.^7^, ^8^

The current report from the ongoing international STAR registry reports bleeding outcomes in patients on ticagrelor or DOAC undergoing emergent OHT with the intraoperative use of a device capable of removing these drugs from circulation. The main observation of this case series of 7 such patients is that this novel strategy employing intraoperative drug removal was associated with low perioperative bleeding rates, no reoperations, and favorable overall outcomes. Interestingly, 24-hour CTD volumes in our analysis of patients on ticagrelor or DOAC undergoing OHT compared favorably to published reports. Specifically, Kedziora et al^9^ reported a mean 24-hour CTD volume of 695 ml in patients undergoing OHT who were not on antithrombotics, which is slightly higher than in the current report. In fact, 2 patients who were dialysis-dependent preoperatively did so well during OHT that they also received a kidney transplant on the first postoperative day. Reassuringly, there were no device-related adverse events reported in any of these patients.

The number of patients on the heart transplant waiting list taking blood thinners is not insignificant.^10^ Of note, reversal agents have been proposed in this setting, and at least 1 case report discussed dabigatran reversal with idarucizumab for OHT.^11^ However, in regard to andexandet alpha, the European Medicines Agency advises against use before heparinization since it causes unresponsiveness to the anticoagulant effects of heparin, a very serious limitation in the setting of CPB.

The availability of a device that can easily be integrated into the CPB circuit and efficiently remove certain antithrombotic drugs from the circulation is an attractive option to mitigate the high risk of severe bleeding with OHT in these patients. The current report suggests low bleeding rates after OHT on patients on ticagrelor or DOAC with the use of the device. The hypothesis of whether ticagrelor removal can reduce serious perioperative bleeding in patients undergoing urgent cardiac surgery is currently formally tested in the pivotal, double-blind, randomized Safe and Timely Antithrombotic Removal-Ticagrelor trial (ClinicalTrials.gov Identifier: NCT04976530).^12^

The current results will require confirmation by larger datasets in OHT patients but already suggest that ticagrelor and DOAC may be more attractive compared to alternative agents for patients requiring antithrombotic treatment while awaiting OHT.

## Limitations

The present report is limited by the small sample size and the lack of a control group; however, it establishes the feasibility of this approach and provides early encouraging outcomes. A second limitation is that platelet function or anti-FXa activity was not measured to quantitate the extent and effect of intraoperative drug removal or to determine the impact of the residual oral antithrombotics following heart transplantation. Future larger trials should analyze the current findings in a randomized or propensity-score-matched fashion.

## Conclusion

The present analysis is, to our knowledge, the first case series in patients on AT undergoing OHT with intraoperative AT removal. These preliminary results suggest that this novel intervention is feasible and could be an effective way of reducing complications from perioperative bleeding. However, larger-scale studies are needed to confirm this.

## Disclosure statement

The STAR registry and the present work were funded by CytoSorbents Inc., Princeton, NJ, USA.

## Declaration of Competing Interest

The authors declare the following financial interests/personal relationships, which may be considered as potential competing interests: The STAR registry was sponsored by CytoSorbents Inc., Princeton, NJ, USA. Robert Storey, Michael Schmoeckel, Matthias Thielmann, and Arjang Ruhparwar received speaker honoraria from CytoSorbents Europe, Berlin, Germany. Marijana Matejic-Spasic, Daniel Wendt, and Efthymios Deliargyris received stock options of CytoSorbents Inc., Princeton, NJ, USA. Marijana Matejic-Spasic, Daniel Wendt, and Efthymios Deliargyris are full-time employees of CytoSorbents.

## References

[1] Ikeda S. (2024). Clinical outcomes and anticoagulation therapy in elderly non-valvular atrial fibrillation and heart failure patients. ESC Heart Fail.

[2] Schoerghuber M. (2024). Platelet inhibitor withdrawal and outcomes after coronary artery surgery: an individual patient data meta-analysis. Eur J Cardiothorac Surg.

[3] Storey R.F. (2025). Early CABG with intraoperative hemoadsorption in patients on ticagrelor: real world data from the international Safe and Timely Antithrombotic Removal (STAR) registry. Cardiovasc Revasc Med.

[4] Tripathi R. (2022). Antithrombotic drug removal from whole blood using Haemoadsorption with a porous polymer bead sorbent. Eur Heart J Cardiovasc Pharmacother.

[5] Hassan K. (2023). Intraoperative ticagrelor removal via hemoadsorption during on-pump coronary artery bypass grafting. JTCVS Open.

[6] Douketis J.D., Spyropoulos A.C. (2024). Perioperative management of patients taking direct oral anticoagulants: a review. JAMA.

[7] Cao D. (2023). Reversal and removal of oral antithrombotic drugs in patients with active or perceived imminent bleeding. Eur Heart J.

[8] Schmoeckel M. (2025). Direct-acting oral anticoagulant removal by intraoperative hemoadsorption in CABG and/or single valve surgery: interim analysis of the International Safe and Timely Antithrombotic Removal (STAR) registry. J Cardiothorac Surg.

[9] Kedziora A. (2016). Impact of postoperative bleeding on short-term outcome in patients after orthotopic heart transplantation: a retrospective cohort study. Ann Transplant.

[10] Frering A. (2025). CytoSorb hemoadsorption of apixaban during cardio-pulmonary bypass for heart transplantation. JHLT Open.

[11] Tralhao A. (2017). Dabigatran reversal with idarucizumab in a patient undergoing heart transplantation: first European report. Thromb J.

[12] Gibson C.M. (2022). Rationale and design of the safe and timely antithrombotic removal - ticagrelor (STAR-T) trial: a prospective, multi-center, double-blind, randomized controlled trial evaluating reductions in postoperative bleeding with intraoperative removal of ticagrelor by the drugsorb-ATR device in patients undergoing cardiothoracic surgery within 48 h from last ticagrelor dose. Am Heart J.

